# Battle against time for innovative cancer treatment: an updated cost-effectiveness analysis of pemigatinib in intrahepatic cholangiocarcinoma

**DOI:** 10.1186/s12962-025-00713-w

**Published:** 2026-02-10

**Authors:** I-Ting Wang, Hsiao-Lin Chen, Wei-Ming Huang, Nai-Jung Chiang, Li-Jiuan Shen, Chen-Han Chueh, Yi-Wen Tsai

**Affiliations:** 1 Institute of Health and Welfare Policy, College of Medicine, National Yang Ming Chiao Tung University, Taipei, Taiwan; 2 Medical AI Development Center, Taipei Veterans General Hospital, Taipei, Taiwan; 3 Department of Oncology, Taipei Veterans General Hospital, Taipei, Taiwan; 4 School of Medicine, College of Medicine, National Yang Ming Chiao Tung University, Taipei, Taiwan; 5 School of Pharmacy, College of Medicine, National Taiwan University, Taipei, Taiwan; 6 Graduate Institute of Clinical Pharmacy, College of Medicine, National Taiwan University, Taipei, Taiwan; 7 Department of Pharmacy, National Taiwan University Hospital, Taipei, Taiwan; 8 Herbert Wertheim School of Public Health, University of California San Diego, San Diego, CA USA; 9 College of Pharmaceutical Science, National Yang Ming Chiao Tung University, Taipei, Taiwan

**Keywords:** Pemigatinib, Targeted therapy, *FGFR2*, Intrahepatic cholangiocarcinoma, Economic evaluation, Cost-effectiveness analysis, Health technology reassessment, Provisional payment, Conditional listing

## Abstract

**Background:**

Advanced intrahepatic cholangiocarcinoma (ICC) is a rare cancer. In 2023, Taiwan’s National Health Insurance Administration granted provisional payment for pemigatinib for patients with advanced ICC and *FGFR2* fusions/rearrangements. Previous analyses of the 2020 FIGHT-202 trial found pemigatinib was not cost-effective; however, updated 2024 data showed promising long-term benefits. This study aimed to reassess the cost-effectiveness of pemigatinib for advanced ICC with *FGFR2* fusions/rearrangements from the perspective of the Taiwanese healthcare payer.

**Methods:**

We used a three-state partitioned survival model to evaluate the lifetime cost-effectiveness of pemigatinib compared to mFOLFOX or 5-FU/LV, based on data from the updated FIGHT-202, ABC-06, and updated NIFTY pivotal trials. Utility, disutility, and cost parameters were derived from published sources. The primary measure was the incremental cost-effectiveness ratio (ICER). Scenario and sensitivity analyses were performed to determine the break-even year and main parameters influencing cost-effectiveness analysis results.

**Results:**

Pemigatinib was found to be cost-effective with high certainty (mFOLFOX: ICER = US$83,475, probability = 81.4%; 5-FU/LV: ICER = US$84,386, probability = 79.9%). The updates to the FIGHT-202 and NIFTY trials significantly increased the expected value of information (mFOLFOX: US$30,405; 5-FU/LV: US$28,851). Although pemigatinib incurred higher cumulative costs initially, the break-even point was reached between 7.6 and 7.7 years. The key factors influencing the cost-effectiveness results were the use of updated FIGHT-202 trial data and a lifetime simulation horizon.

**Conclusion:**

Pemigatinib is cost-effective with updated long-term benefits from the pivotal trial in the lifetime simulation for advanced patients with advanced ICC and *FGFR2* fusions/rearrangements. Given the rarity of advanced ICC, applying updated pivotal trial data with local real-world data for cost-effectiveness reassessment can be an efficient way under Taiwan’s Provisional Payment scheme.

**Supplementary Information:**

The online version contains supplementary material available at 10.1186/s12962-025-00713-w.

## Background

Intrahepatic cholangiocarcinoma (ICC) is a rare cancer often diagnosed in advanced stages owing to its asymptomatic nature, contributing to rising global mortality rates [1, 2]. Notably, Taiwan has observed a concerning upward trend in the age-adjusted incidence rate over the past two decades, rising from 2.21 per 100,000 in 2000 to 3.14 per 100,000 in 2022, underscoring the urgent need for effective treatments in the local context [3]. International guidelines recommend systemic therapy for advanced ICC [4–6]. The National Comprehensive Cancer Network recommends pemigatinib, an FGFR kinase inhibitor, as a second-line treatment for advanced ICC with *FGFR2* fusions/rearrangements and modified FOLFOX (a combination of oxaliplatin, folinic acid, and fluorouracil [5-FU]) for those without *FGFR2* alterations [4]. In 2020, the U.S. Food and Drug Administration accelerated approved pemigatinib based on the 20-month FIGHT-202 Phase II trial [7]. In June 2024, the updated 50-month data from the FIGHT-202 trial confirmed pemigatinib’s efficacy, demonstrating improved durable responses and prolonged overall survival (OS) compared to the 2020 results [8].

To facilitate early access to high-cost innovative medicines, the UK's National Institute for Health and Care Excellence (NICE) introduced Managed Access Agreements [9], alongside health technology reassessments (HTR) for permanent reimbursement decisions. Inspired by this model, Taiwan’s National Health Insurance Administration (NHIA) launched two key initiatives. First, in 2023, the Provisional Payment scheme was introduced to provide early access to new drugs with unmet medical needs that have not yet completed Phase 3 trials. This scheme provisopnally covers drugs for two to three years (up to a maximum of five) and mandates the collects real-world data (RWD) to inform subsequent HTR decisions. Second, in 2025, Taiwan launched the Cancer Drug Fund and Conditional Listing scheme. This initiative targets cancer drugs that have completed Phase 3 trials but are not yet covered due to global budget constraints, as well as innovative oncology treatments deemed necessary by the NHIA. Under this scheme, coverage is limited to a duration of two to three years (up to a maximum of five). Pharmaceutical companies must submit a reassessment report before the conditional listing period ends to facilitate the decision on permanent listing.

Pemigatinib, based on preliminary efficacy data from the 2020 FIGHT-202 trial, was the first drug approved under the Provisional Payment scheme for second-line treatment of advanced ICC with *FGFR2* fusions/rearrangements. A 5-year cost-effectiveness analysis (CEA) conducted prior to reimbursement indicated that pemigatinib’s value-based price of NT$8,910 (US$286.04) per 13.5 mg was lower than the NHIA’s listing price of NT$12,500 (US$401.28), suggesting that the current listing price exceeds the estimated value-based price, making pemigatinib not cost-effective compared to systemic therapies with mFOLFOX or 5-FU/LV over 5 years [10].

The NHIA’s Provisional Payment scheme requires a reassessment of pemigatinib’s cost-effectiveness and decision uncertainty for permanent reimbursement two to five years after initial coverage. Given that advanced ICC is a rare cancer, local RWD may hardly be insufficient for HTR within this timeframe. Updating trial findings and extending the time horizon will provide valuable insights for future policy. This study aims to update the cost-effectiveness of pemigatinib as second-line therapy compared to mFOLFOX and 5-FU/LV for advanced ICC with *FGFR2* fusions/rearrangements, using the latest 2024 efficacy data from the FIGHT-202 trial. It also analyzes the key parameters affecting the cost-effectiveness results and the decision-making risks associated with uncertainty.

## Methods

### Cost-effectiveness framework

**Fig. 1:**
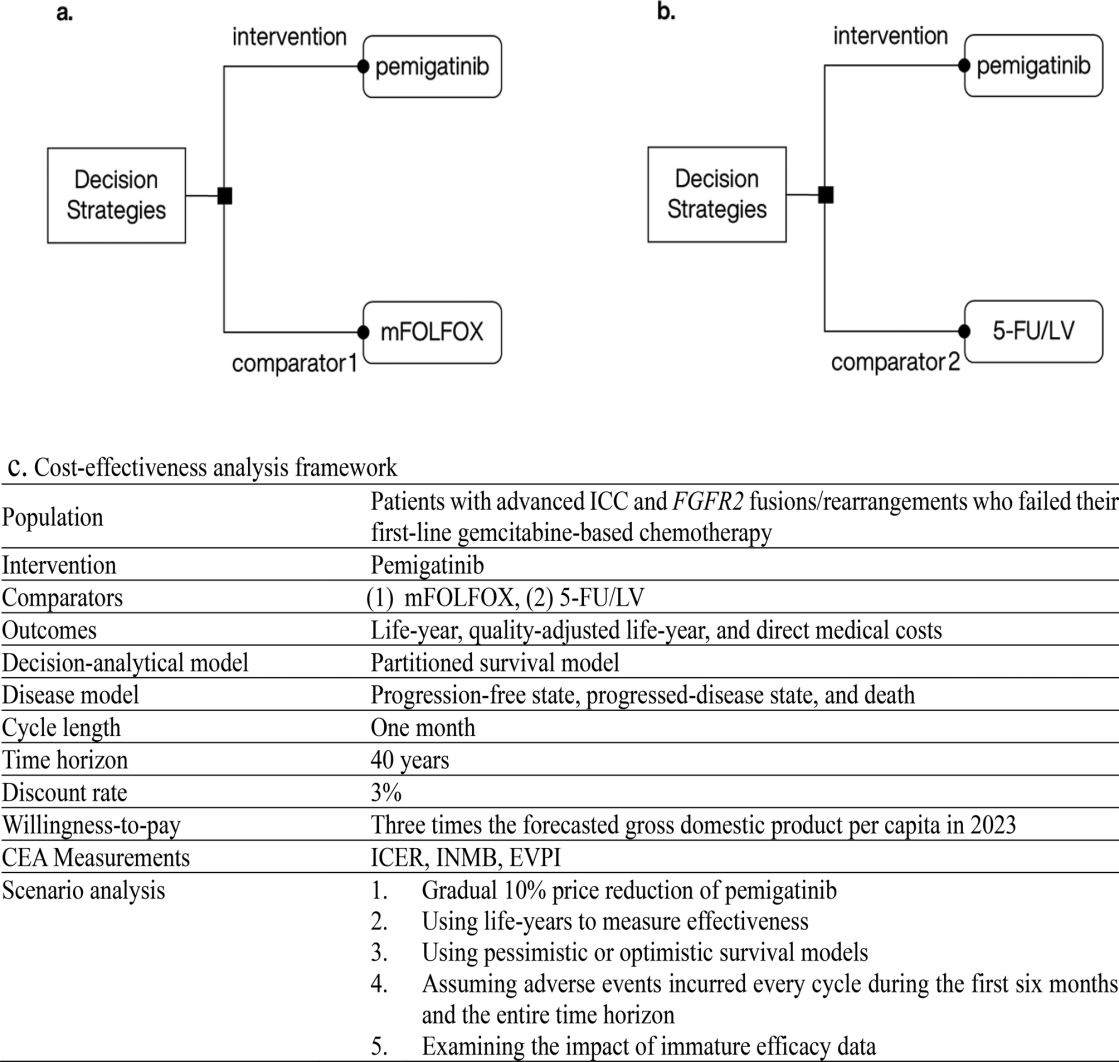
Two decision problems and cost-effectiveness analysis: intervention regimen of pemigatinib versus comparator 1 of mFOLFOX (**a**), intervention regimen of pemigatinib versus comparator 2 of 5-FU/LV (**b**), and the cost-effectiveness analysis framework (**c**) *ICC* intrahepatic cholangiocarcinoma; *FGFR2* fibroblast growth factor receptor 2; *mFOLFOX* a combination of oxaliplatin, folinic acid, and fluorouracil; *5-FU/LV* fluorouracil and leucovorin; *CEA* cost-effectiveness analysis; *ICER* incremental cost-effectiveness ratio; *INMB* incremental net monetary benefit; *EVPI* expected value of perfect information

**Fig. 2:**
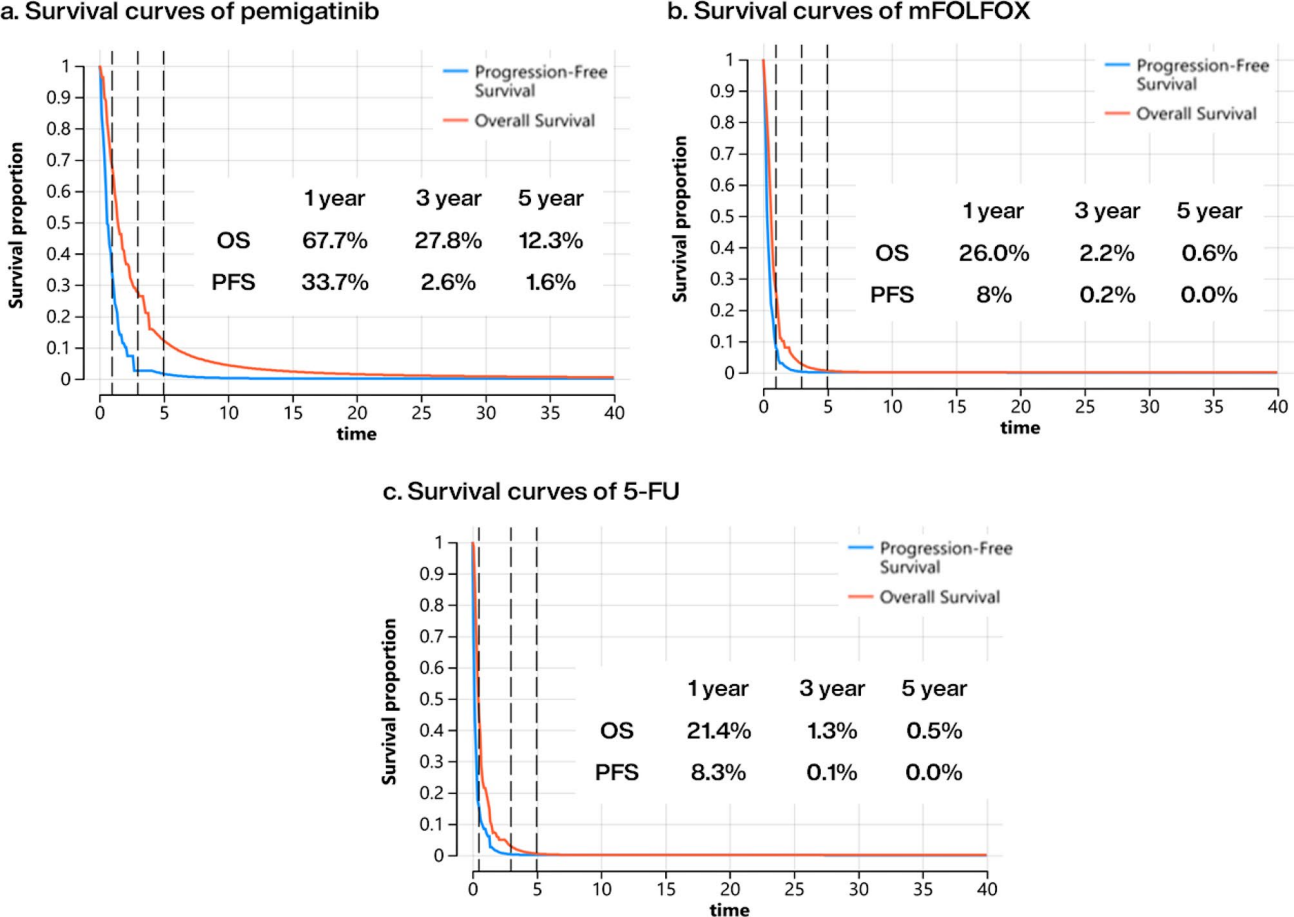
Survival benefits, cumulated cost, and break-even year analyses in pemigatinib versus mFOLFOX and 5-FU/LV: progression-free survival and overall survival curve of pemigatinib (**a**), mFOLFOX (**b**), and 5-FU/LV (**c**). The black dotted lines present the first, third, and fifth years for survival extrapolation. *mFOLFOX* a combination of oxaliplatin, folinic acid, and fluorouracil; *5-FU/LV* fluorouracil and leucovorin; *INMB* incremental net monetary benefit; *NT$* New Taiwan dollars; *OS* overall survival; *PD* progressed disease; *PF* progression-free; *PFS* progression-free survival; *QALY* quality-adjusted life-year

From the perspective of Taiwan’s NHIA, we evaluated the cost-effectiveness of pemigatinib compared with two regimens: (1) mFOLFOX, following international guidelines (Fig. 1a), and (2) 5-FU/LV, covered by Taiwan National Health Insurance (NHI, Fig. 1b). The CEA model (Fig. 1c) used three-state partitioned survival models (PSM) to estimate the proportions in three health states—progression-free (PF), progressed disease (PD), and death—over a 40-year horizon. Health outcomes and direct medical costs were discounted at an annual rate of 3%, in accordance with the local pharmacoeconomic guideline [11]. The willingness-to-pay (WTP) threshold was set at three times the forecasted 2023 gross domestic product (GDP) per capita (NT$3,023,055/US$97,048) [12] per WHO recommendations [13, 14]. The model was developed in TreeAge Pro Healthcare (version 2023 R1, TreeAge Software, LLC). The study followed the reporting guidelines of the Professional Society of Pharmacoeconomics and Outcomes Research and the consolidated health economic evaluation standards [15].

### Population

The study population consisted of patients with advanced ICC and *FGFR2* fusions/rearrangements. Inclusion criteria, consistent across the FIGHT-202 [8, 16], ABC-06 [17], and NIFTY trials [18, 19], included adults aged ≥ 18 years (≥ 19 in NIFTY), with histologically or cytologically confirmed disease progression after at least one prior systemic therapy (excluding selective FGFR inhibitors in FIGHT-202), and a good ECOG performance status (0–1 in NIFTY and ABC-06; 0–2 in FIGHT-202). Patients in the pemigatinib arm differed from those in the other two arms at baseline. The pemigatinib group was younger (mean age 56 years, range 26–77) [8, 16] compared to the mFOLFOX (mean age 65 years, range 59–72) [17] and 5-FU/LV arms (mean age 65 years, range 37–80) [18, 19]. The proportion of females was higher in the pemigatinib arm (61%) than in the mFOLFOX (47%) and 5-FU/LV arms (44%). Since there is no evidence suggesting a gender-based difference in ICC prognosis [20], gender differences are not expected to affect efficacy.

### Intervention and comparators

The treatment strategies for the intervention and comparators were based on established treatment protocols from three clinical trials. The intervention involved pemigatinib, administered at 13.5 mg daily dose every 3 weeks (2 weeks on and 1 week off), based on the FIGHT-202 trial [8, 16]. Comparator 1 was mFOLFOX (oxaliplatin at 85 mg/m², L-folinic acid 175 mg or folinic acid 350 mg, 5-FU 400 mg/m² bolus, and 5-FU 2,400 mg/m² continuous intravenous infusion for 46 h every 2 weeks) from the ABC-06 trial [17]. Comparator 2 was 5-FU/LV (leucovorin 400 mg/m² and 5-FU 2,400 mg/m² every 2 weeks) from the NIFTY trial [18, 19]. All treatment strategies were assumed to continue until disease progression, after which all patients would receive standardized supportive care.

### Cost-effectiveness measures

We estimated the incremental cost-effectiveness ratio (ICER) and incremental net monetary benefit (INMB) as measures of cost-effectiveness. The ICER represents the incremental cost per unit of incremental effectiveness of pemigatinib compared to two comparators: mFOLFOX (comparator 1) and 5-FU/LV (comparator 2). These ICER values were evaluated against the WTP threshold. The INMB quantifies the net monetary benefit of pemigatinib after accounting for the incurred incremental costs.

### Effectiveness

In the absence of published indirect comparisons and accessible individual patient data (IPD), baseline adjustment between the intervention and comparator regimens was not feasible; therefore, naïve indirect comparisons were conducted. To estimate survival functions, we used WebPlotDigitizer (version 4.7) [21] to extract data points from the Kaplan–Meier (KM) curves in the updated FIGHT-202 [8] and NIFTY trials [19]. Using the IPDfromKM package [22] in R software (version 4.3.2, Vienna, Austria, R Foundation for Statistical Computing), we generated pseudo-IPD and compared the reconstructed OS and progression-free survival (PFS) plots with those in the published pivotal trials. We fitted the pseudo-IPD into standard parametric models (exponential, gamma, generalized gamma, Gompertz, Weibull, log-logistic, and log-normal distributions) according to NICE guidance [23]. The primary survival models for extrapolation were selected based on visual assessment, the Akaike information criterion (AIC), the Bayesian information criterion (BIC), and expert opinion (AIC and BIC values are presented in the Supplementary Table 1). For each treatment arm, we constructed hybrid PFS and OS curves by combining the KM curves observed during the trial period with extrapolated parametric curves extended to a 40-year lifetime horizon. For the mFOLFOX arm, as the ABC-06 trial has not reported updated results since 2022, we derived effectiveness estimates from our previous CEA [10] to ensure comparability. In our base-case analysis, log-normal and log-logistic distributions were selected for the PFS and OS curves in the pemigatinib arm, respectively. Log-normal distributions were applied to both the PFS and OS curves for the mFOLFOX and 5-FU/LV arms.

### Direct medical cost

The direct medical costs related to the PF state include both medication and non-medication expenses reimbursed by the NHIA. The unit price for pemigatinib was US$401 per 13.5 mg (US$97,645 annually). The medication costs for mFOLFOX and 5-FU/LV were US$13,230 and US$5,707 per year, respectively [10, 24]. The non-medication costs for pemigatinib were assumed to be the same as for mFOLFOX and 5-FU/LV, based on literature sources [10, 24].

The direct medical costs associated with the PD state primarily consist of supportive care expenses covered by the NHIA, totaling US$15,978 per year [10, 24]. These costs were assumed to be the same across both the intervention and comparator arms. We assumed that treatment-related adverse effects (AEs) occur only during the PF state and thus do not impact supportive care costs in the PD state. These costs are reported in 2023 U.S. dollars (1 USD = 31.35 TWD) [25].

Taiwan’s NHIA quantifies health services using a point-based fee schedule. Under the NHIA’s global budget system, the monetary value of each point is NT$1 for medication, while the value for non-medication services is adjusted by a conversion factor. In 2023, the conversion factor was 0.9198 [26], and it is adjusted annually and varies by region. All costs, except for pemigatinib, were assumed to follow gamma distributions, while the conversion factor was assumed to follow a uniform distribution.

### Utilities

As in previous cost-effectiveness studies for advanced ICC [10, 24, 27], we adopted utility parameters from patients with advanced hepatocellular carcinoma receiving sorafenib, setting utility values at 0.76 for the PF state and 0.68 for the PD state [28]. Disutility values for adverse events were sourced from studies on metastatic renal carcinoma and a prior hepatocellular carcinoma study, with disutility values set at 0.16 for adverse events [29] and 0.025 for subcutaneous or intravenous therapies [30, 31]. Both utility and disutility values were assumed to follow a beta distribution.

### Adverse events

To be conservative, we calculated the probability of AEs (≥ grade 3) during the trial period by summing occurrences of each AE (≥ grade 3) in each trial. The probabilities were 56% for pemigatinib [16], 54% for mFOLFOX [17], and 18% for 5-FU/LV [18]. In our base-case analysis, we assumed AEs occurred during the initial cycle in the PF state, and we varied this assumption in the scenario analysis.

### Sensitivity analyses

We conducted deterministic sensitivity analysis (DSA) and probabilistic sensitivity analysis (PSA) to assess uncertainties in the base-case results. In the DSA, each parameter varied within its 95% confidence interval or by ± 25% from the baseline, and the conversion factor ranged between its 2023 maximum and minimum. For the PSA, we performed a Monte Carlo simulation with 1,000 iterations using predefined distributions for each parameter to estimate cumulative cost and quality-adjusted life years (QALYs) over a 40-year horizon. Results were displayed on a cost-effectiveness plane, with cost-effectiveness acceptability curves (CEAC) and the expected value of perfect information (EVPI) calculated. Additionally, we conducted a break-even-year analysis to assess changes in INMB over time.

### Scenario analyses

Scenario analyses were conducted in two parts. The first part assessed the impact of varying efficacy models, parameters, and assumptions on the base-case CEA results. For efficacy, we defined the optimistic scenario with the best extrapolation of PFS and OS curves for pemigatinib (log-logistic distributions) and the pessimistic scenario with the worst extrapolation (Weibull distributions). Additional scenarios included a 10% price reduction, outcomes measured in life years, time horizons of 2, 5, 10, 20, and 30 years, and differing AE durations (occurring every cycle during the first 6 months in the PF state, and another lasting the entire time horizon). In addition, we conducted a scenario analysis incorporating AE-specific disutilities. We selected adverse events with an incidence greater than 5% and applied event-specific values, consistent with the approach outlined in the pemigatinib NICE health technology assessment report [32] and a previous study [33] (detailed disutility values are presented in Table 1).

**Table 1:** Model parameters. Baseline values, ranges, and distributions for sensitivity analyses

Parameters and distributions	Estimated value	DSA		PSA	Source
		Range (± 25%)		Distribution	
**1. Overall survival**					
Pemigatinib					
Weibull (Fight-202, 2020)	Scale: 22.065	17.486	27.843	Normal (22.065, 2.619)	[16]
	Shape: 1.536	1.184	1.993	Normal (1.536, 0.204)	
Log-logistic (Fight-202, 2024)	Scale: 18.387	14.919	22.661	Normal (18.387, 1.961)	[8]
	Shape: 1.626	1.384	1.961	Normal (1.626, 0.156)	
mFOLFOX: log-normal	Ln(mean): 1.834	1.642	2.023	Normal (1.834, 0.098)	[17]
	Ln(SD): 0.873	0.742	1.027	Normal (0.873, 0.072)	
5-FU/LV					
Log-normal (NIFTY, 2021)	Ln(mean): 1.764	1.586	1.943	Normal (1.764, 0.091)	[18]
	Ln(SD): 0.816	0.691	0.965	Normal (0.816, 0.07)	
Log-normal (NIFTY, 2023)	Ln(mean): 1.794	1.618	1.970	Normal (1.794, 0.090)	[19]
	Ln(SD): 0.810	0.691	0.949	Normal (0.810, 0.066)	
**2. Progression-free survival**					
Pemigatinib					
Log-normal (Fight-202, 2020)	Ln(mean): 1.962	1.76	2.164	Normal (1.962, 0.103)	[16]
	Ln(SD): 0.971	0.82	1.15	Normal (0.971, 0.084)	
Log-normal (Fight-202, 2024)	Ln(mean): 1.988	1.792	2.185	Normal (1.988, 0.100)	[8]
	Ln(SD): 0.989	0.852	1.149	Normal (0.989, 0.075)	
mFOLFOX: log-normal	Ln(mean): 1.430	1.265	1.595	Normal (1.43, 0.084)	[17]
	Ln(SD): 0.754	0.643	0.885	Normal (0.754, 0.062)	
5-FU/LV					
Log-normal (NIFTY, 2021)	Ln(mean): 0.8	0.57	1.031	Normal (0.8, 0.118)	[18]
	Ln(SD): 1.067	0.91	1.251	Normal (1.067, 0.087)	
Log-normal (NIFTY, 2023)	Ln(mean): 0.809	0.615	1.004	Normal (0.809, 0.099)	[19]
	Ln(SD): 0.9209	0.791	1.072	Normal (0.921, 0.072)	
**3. Optimistic survival scenario**					
Overall survival:	Scale: 18.387	14.919	22.661	Normal (18.387, 1.961)	[8]
Pemigatinib: Log-logistic	Shape: 1.626	1.384	1.961	Normal (1.626, 0.156)	
Progression-free survival:	Scale: 7.512	6.166	9.153	Normal (7.512, 0.757)	[8]
Pemigatinib: Log-logistic	Shape: 1.736	1.461	2.061	Normal (1.736, 0.152)	
**4. Pessimistic survival scenario**					
Overall survival:	Scale: 0.019	0.009	0.041	Normal (0.019, 0.007)	[8]
Pemigatinib: Weibull PH	Shape: 1.194	0.991	1.439	Normal (1.194, 0.114)	
Progression-free survival:	Scale: 0.057	0.033	0.097	Normal (0.057, 0.015)	[8]
Pemigatinib: Weibull PH	Shape: 1.177	1.006	1.377	Normal (1.177, 0.094)	
**5. Genetic testing fee (USD)**	934	701	1,168	Fixed	Market price
**6. Medication cost (per year**,** USD)**					
Pemigatinib	97,645	73,235	122,057	Uniform (73,235, 122,057)	NHIA listing price
mFOLFOX	13,230	9,922	16,536	Gamma (41.39, 0.003115)	[10, 24]
5-FU/LV	5,707	4,280	7,133	Gamma (36.83, 0.0065415)	[10, 24]
**7. Nonmedication cost (per year**,** USD)**					
Pemigatinib	9,361	7,020	11,701	Gamma (16.56, 0.001869)	[10, 24]
Chemotherapy	27,512	20,634	34,389	Gamma (59.94, 0.0021805	[10, 24]
**8. Supportive care cost** **(per year**,** USD)**	15,978	11,983	19,972	Gamma (69.3, 0.004361)	[10, 24]
**9. Utility**					
Progression-free state	0.760	0.57	0.95	Beta (4.7, 1.5)	[28]
Progressed disease state	0.680	0.51	0.85	Beta (29, 13.6)	[28]
**10. Disutility**					
Grade 3 and higher AEs	0.160	0.120	0.200	Beta (36, 193)	[29]
Intravenous therapy	0.025	0.019	0.031	Fixed	[30, 31]
Stomatitis	0.0375			Fixed	[32]
Palmar-plantar erythrodysesthesia	0.085			Fixed	[32]
Hypophosphatemia	0			Fixed	[32]
Fatigue or lethargy	0.085			Fixed	[32]
Neutropenia	0.0607			Fixed	[32]
Infection	0.085			Fixed	[32]
Pain	0.069			Fixed	[32]
Biliary event	0.085			Fixed	[32]
Hypertension	0.03			Fixed	[33]
**11. Probability of AE**					
Pemigatinib					
Stomatitis	8.3%			Fixed	[8]
Palmar-plantar erythrodysesthesia	5.6%			Fixed	[8]
Fatigue or lethargy	1.9%			Fixed	[8]
Hypophosphatemia	10.2%			Fixed	[8]
mFOLFOX					
Hypophosphatemia	1%			Fixed	[17]
Fatigue or lethargy	19%			Fixed	[17]
Neutropenia	12%			Fixed	[17]
Infection	18%			Fixed	[17]
Pain	10%			Fixed	[17]
Biliary event	19%			Fixed	[17]
Hypertension	5%			Fixed	[17]
5-FU/LV					
Stomatitis	5%			Fixed	[19]
Palmar-plantar erythrodysesthesia	4%			Fixed	[19]
Hypophosphatemia	7%				[19]
**11. Discount rate (per year)**	0.03	0.000	0.050	Fixed	[11]

Note: Costs listed in 2023 US dollars

*5-FU/LV fluorouracil and leucovorin; AE adverse event; DSA* deterministic sensitivity analysis; *FGFR2* fibroblast growth factor receptor 2; *mFOLFOX* a combination of oxaliplatin, folinic acid, and fluorouracil; *PH* proportional hazards; *PSA* probabilistic sensitivity analysis; *NHIA* National Health Insurance Administration

The second part evaluated the impact of using complete versus immature efficacy data on CEA results. We used the methodology from a prior study [10] as our baseline framework, aligning the WTP threshold, medication price, and time horizon with those in the reference case. Based on this reference case, we then updated the efficacy data for 5-FU/LV and pemigatinib to examine how the updated efficacy data influenced the CEA results.

### Model validation

To validate our model, we used the Assessment of the Validation Status of Health-Economic Decision Models [34]. Our conceptual model followed the approaches outlined by NICE [32] and the Canadian Agency for Drugs and Technologies in Health [35]. However, due to the unavailability of IPD from pivotal trials, a direct comparison with a Markov model was not feasible. To ensure model accuracy, four researchers conducted a comprehensive review using TreeAge Pro Healthcare and R to detect and correct any logical errors.

## Results

### Base-case analysis

Based on the updated efficacy data from pivotal trials, the base-case CEA results over a lifetime (Table 2) showed that compared to mFOLFOX, pemigatinib resulted in an incremental QALY of 1.20, additional costs of US$99,762, and an ICER of US$83,475 per QALY. Compared to 5-FU/LV, pemigatinib had an incremental QALY of 1.27, additional costs of US$107,186, and an ICER of US$84,386 per QALY. Both ICERs were below the WTP threshold (US$97,048), with INMB values of US$16,221 (mFOLFOX) and US$16,083 (5-FU/LV). These results indicate the cost-effectiveness of pemigatinib over a lifetime.

**Table 2:** Base-case results

	Outcomes of partitioned survival models			Incremental changes	
Treatment strategy	Intervention pemigatinib	Comparator 1 mFOLFOX	Comparator 2 5-FU/LV	Pemigatinib vs. mFOLFOX	Pemigatinib vs. 5-FU/LV
**Cost**	122,612	22,850	15,426	99,762	107,186
Total cost of the PF state	99,702	17,661	9,591	82,041	90,111
Medication cost	91,537	6,028	1,748	85,509	89,788
Nonmedication cost	8,165	11,633	7,842	-3,468	323
Total cost of the PD state	22,909	5,189	5,834	17,720	17,074
**LY**					
PF state	0.94	0.46	0.25	0.48	0.69
Overall	2.49	0.81	0.64	1.68	1.85
**QALY**					
PF state	0.71	0.33	0.22	0.38	0.48
Overall	1.76	0.57	0.49	1.20	1.27
**ICER**					
Incremental cost per LY gained				59,225	57,936
Incremental cost per QALY gained				83,475	84,386
**INMB**					
LY				63,712	72,360
QALY				16,221	16,083
**EVPI/person**				1,954	2,313

Note: Costs listed in 2023 US dollars

*mFOLFOX* a combination of oxaliplatin, folinic acid, and fluorouracil; *5-FU/LV fluorouracil and leucovorin; EVPI expected value of perfect information; ICER incremental cost-effectiveness ratio; INMB* incremental net monetary benefit; *LY* life-year; *PD* progressed disease; *PF* progression free; *QALY* quality-adjusted life-year

Figure 2 present that pemigatinib had significantly superior PFS probabilities compared to mFOLFOX or 5-FU/LV. In the first year, the PFS probability was 33.7% for pemigatinib, compared to 8% for mFOLFOX and 8.3% for 5-FU/LV. By the third year, the PFS probability for pemigatinib was 2.6%, whereas all patients receiving mFOLFOX or 5-FU/LV had either progressed or passed away. Regarding PD states, pemigatinib showed superior efficacy over the first 3 years in OS and exhibited longer tail survival probabilities for OS and PFS.

**Fig. 3:**
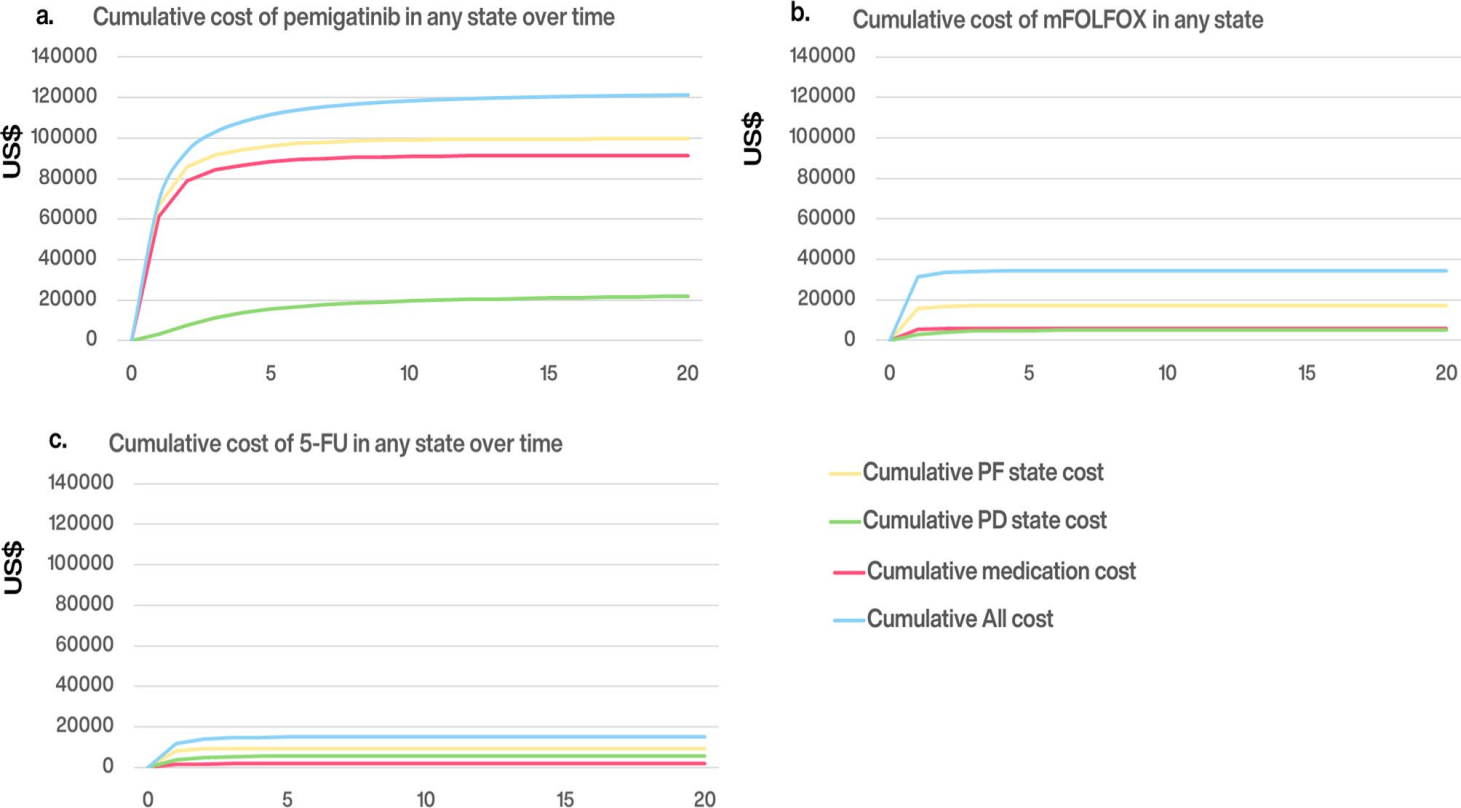
Cumulative cost over time in the strategy of pemigatinib (**a**), mFOLFOX (**b**), and 5-FU/LV (**c**). Costs are listed in 2023 US dollars. *mFOLFOX* a combination of oxaliplatin, folinic acid, and fluorouracil; *5-FU/LV* fluorouracil and leucovorin; *INMB* incremental net monetary benefit; *NT$* New Taiwan dollars; *OS* overall survival; *PD* progressed disease; *PF* progression-free; *PFS* progression-free survival; *QALY* quality-adjusted life-year

Figure 3 compare the cumulative medical costs of pemigatinib with mFOLFOX and 5-FU/LV over time. Owing to faster progression and higher mortality, the cumulative medical costs for mFOLFOX and 5-FU/LV plateau within 3 years. In contrast, pemigatinib’s cumulative costs during PF state increase rapidly over the first 5 years. This rise is driven by improved survival outcomes along with high medication costs, with 90% of the PF costs attributable to pemigatinib's medication cost. After the disease progressed, the increase in medical costs during the PD state had a relatively marginal effect on overall costs.

**Fig. 4:**
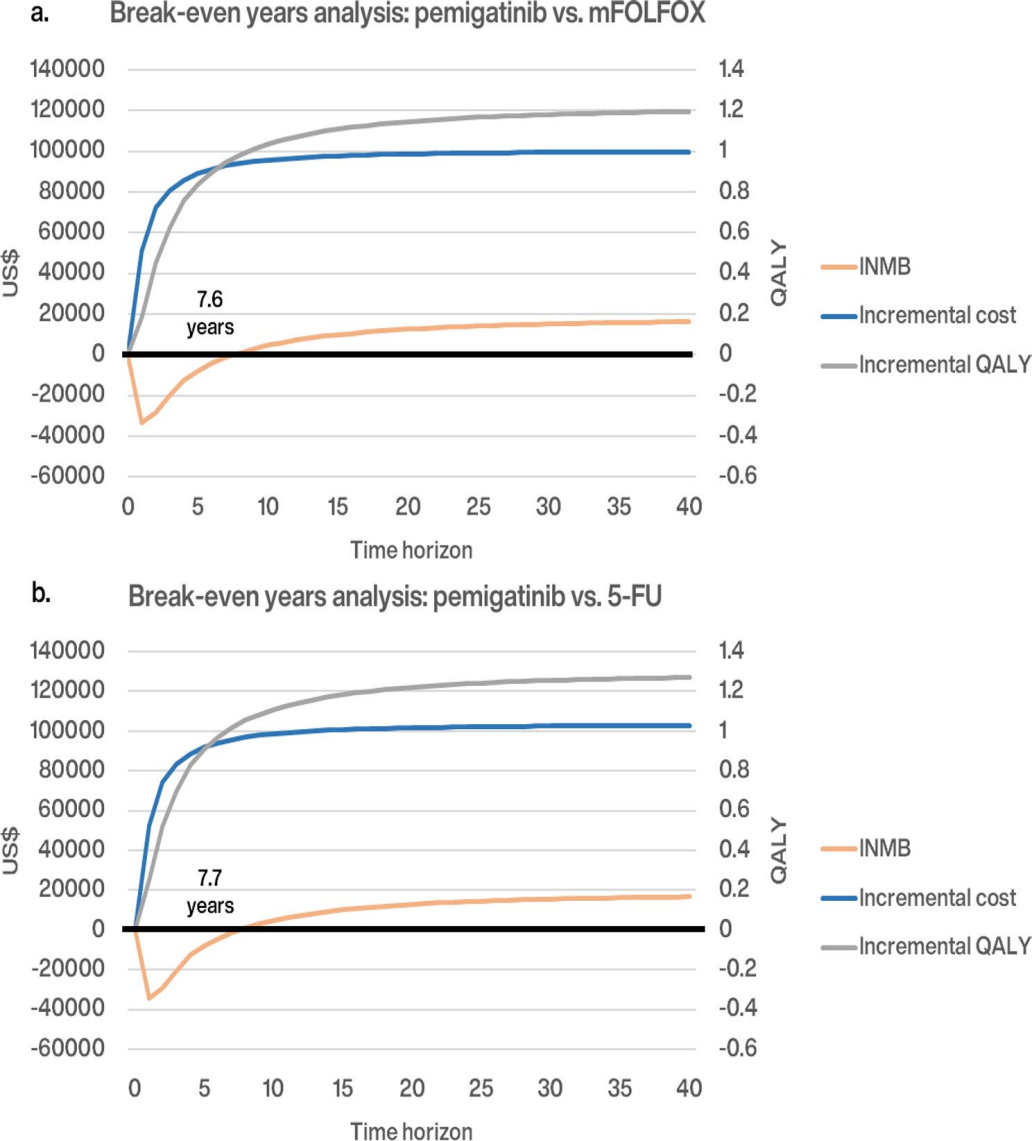
Breakeven years analysis results of pemigatinib versus mFOLFOX (**a**) and 5-FU/LV (**b**). Costs are listed in 2023 US dollars. The black dotted lines present the first, third, and fifth years for survival extrapolation. The black solid lines indicate the break-even year. *mFOLFOX* a combination of oxaliplatin, folinic acid, and fluorouracil; *5-FU/LV* fluorouracil and leucovorin; *INMB* incremental net monetary benefit; *NT$* New Taiwan dollars; *OS* overall survival; *PD* progressed disease; *PF* progression-free; *PFS* progression-free survival; *QALY* quality-adjusted life-year

Figure 4 present that the incremental cumulative medical cost of pemigatinib stabilizes after 7 years as marginal costs decrease as patients either progress or pass away. The high medical cost of pemigatinib was offset by the cumulative incremental QALYs within 8 years, reaching breakeven in 7.6 years for mFOLFOX and 7.7 years for 5-FU/LV. This indicates that pemigatinib becomes cost-effective by sustaining patient survival at a low marginal cost in the PD state over the long term.

### Base-case sensitivity analysis

**Fig. 5:**
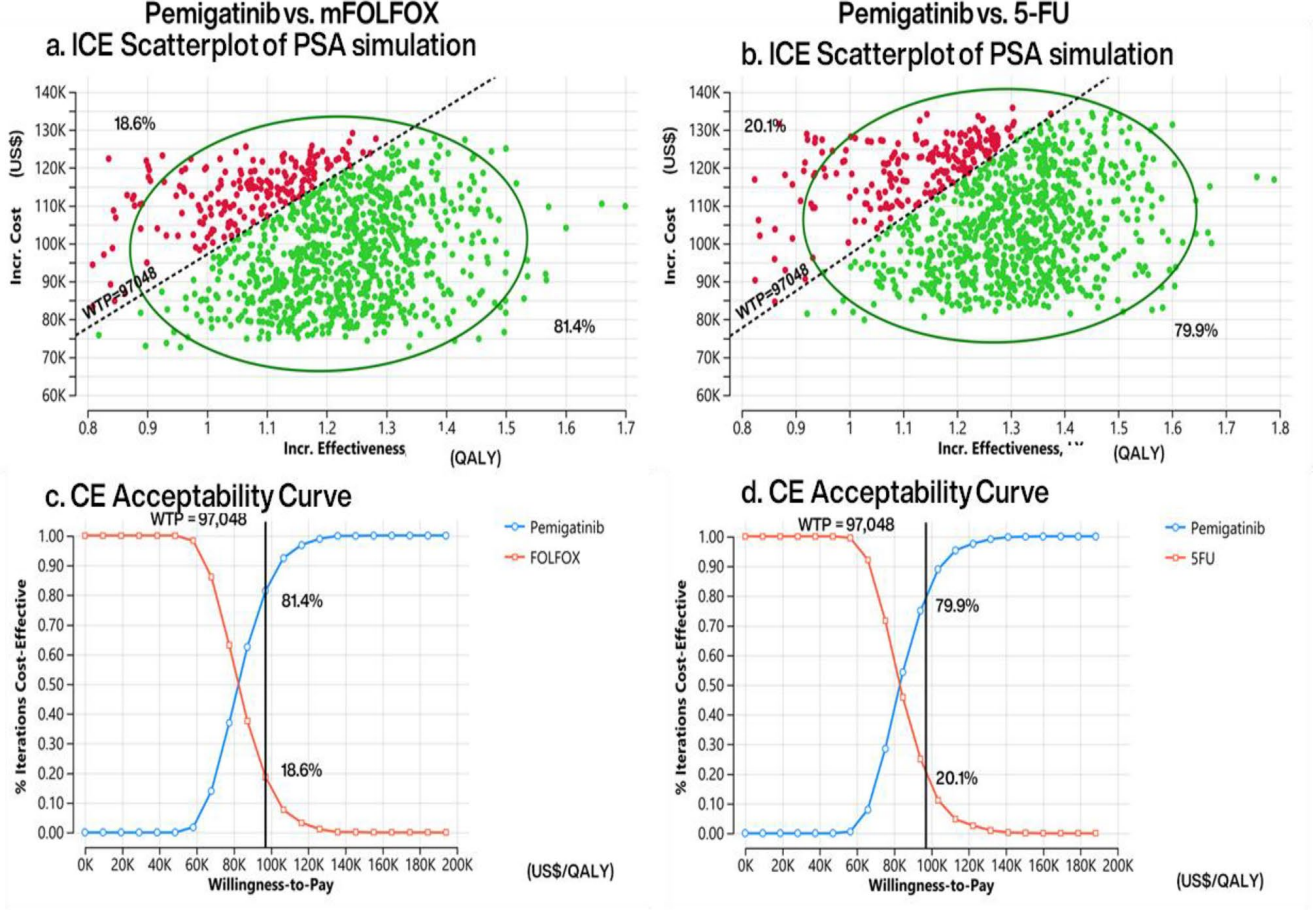
Probabilistic sensitivity analysis results: incremental cost-effectiveness plane for pemigatinib versus mFOLFOX (**a**) and 5-FU/LV (**b**), cost-effectiveness acceptance curve for pemigatinib versus mFOLFOX (**c**) and 5-FU/LV (**d**). 5-*FU/LV* fluorouracil and leucovorin; *CE* cost-effectiveness; *ICE* incremental cost-effectiveness; *Incr*. incremental; *mFOLFOX* a combination of oxaliplatin, folinic acid, and fluorouracil; *PSA* probabilistic sensitivity analysis

**Fig. 6:**
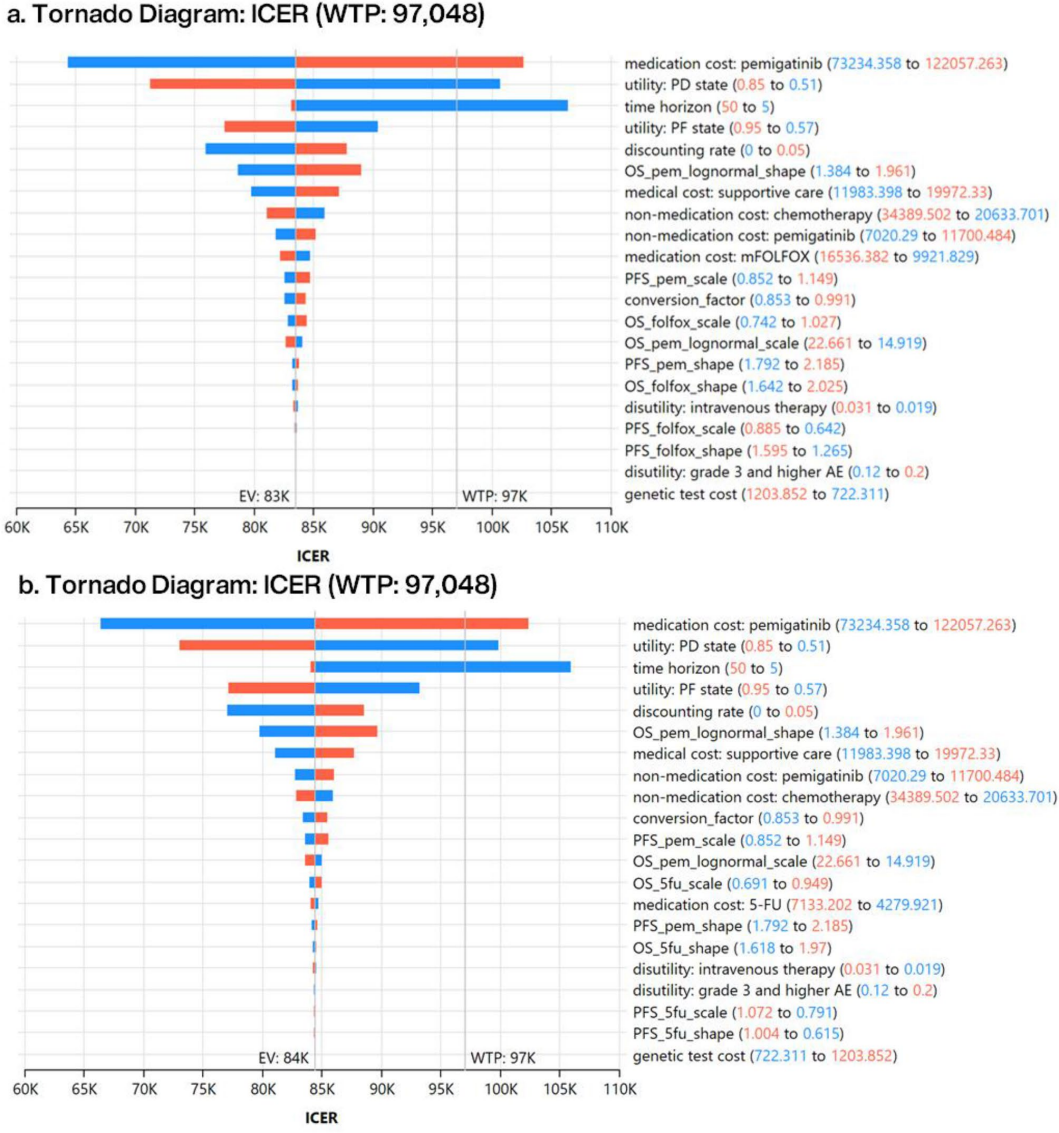
Deterministic sensitivity analysis results: drivers of incremental cost per quality-adjusted life-year (QALY) gained for pemigatinib versus mFOLFOX (**a**) and 5-FU/LV (**b**). Costs are listed in 2023 US dollars. The black lines indicate the willingness-to-pay threshold of US$97,048 per QALY gained. *WTP* willingness to pay; *EV* expected value; *ICER* incremental cost-effectiveness ratio; *mFOLFOX* a combination of oxaliplatin, folinic acid, and fluorouracil; *5-FU/LV* fluorouracil and leucovorin; *PD* progressed disease; *PF* progression-free; *OS_pem_lognormal_shape* shape parameter of overall survival (pemigatinib); *OS_pem_lognormal_scale* scale parameter of overall survival (pemigatinib); *PFS_pem_scale* scale parameter of progression-free survival (pemigatinib); *OS_folfox_scale* scale parameter of overall survival (mFOLFOX); *PFS_pem_shape* shape parameter of progression-free survival (pemigatinib); *OS_folfox_shape* shape parameter of overall survival (mFOLFOX); *PFS_folfox_scale* scale parameter of progression-free survival (mFOLFOX); *PFS_folfox_shape* shape parameter of progression-free survival (mFOLFOX); *AE* adverse event; *OS_5fu_scale* scale parameter of overall survival (5-FU/LV); *OS_5fu_shape* shape parameter of overall survival (5-FU/LV); *PFS_5fu_scale* scale parameter of progression-free survival (5-FU/LV); *PFS_5fu_shape* shape parameter of progression-free survival (5-FU/LV)

The Monte Carlo simulation of the PSA (Fig. 5a and b) with 1,000 iterations demonstrated that pemigatinib increased expected QALYs and total costs in both comparators, with most simulated estimates of ICERs under the WTP threshold (green area). The CEACs (Fig. 5c and d) indicated that, at a WTP threshold of US$97,048 per QALY (the black vertical line), pemigatinib had a cost-effectiveness probability of 81.4% compared to mFOLFOX and 79.9% compared to 5-FU/LV (blue line). The tornado diagrams of the DSA (Fig. 6) identified the medication cost of pemigatinib, utilities in the PD state, and the time horizon as the most influential factors, potentially resulting in an ICER exceeding the WTP in both CEA models. Specifically, pemigatinib would no longer be cost-effective if its acquisition cost exceeded US$114,950 compared to mFOLFOX, or US$114,597 compared to 5-FU/LV. Additionally, pemigatinib was not cost-effective when the utility value of the PD state dropped below 0.54. The estimated EVPI for pemigatinib was US$1,954 per person against mFOLFOX and US$2,313 per person against 5-FU/LV (Table 2).

### Scenario analyses

Scenario analysis 1 examined varying economic model components and assumptions (Table 3). Scenario 1.2, focused on life-years without quality adjustment, showed pemigatinib as cost-effective without uncertainty. In an optimistic efficacy model (Scenario 1.3), pemigatinib remained cost-effective with a 5% increase in uncertainty, while in the pessimistic efficacy model (Scenario 1.4), pemigatinib was not cost-effective, with a 31.8–34.3% increase in uncertainty. Including a 10% reduction in pemigatinib cost (Scenario 1.5) resulted in ICERs close to the base case but with increased uncertainty by 13.4–15.1%. Two short-term CEA analysis scenarios (1.6 and 1.7) significantly increased ICER and uncertainty for both comparators. In contrast, scenarios with longer time horizons of 10 years or more (1.8, 1.9, and 1.10) achieved cost-effectiveness. When AEs were disaggregated (scenario 1.13), the difference in the probability of cost-effectiveness was less than 1%.

**Table 3:** Scenario and sensitivity analyses: impact of alternative efficacy models and assumptions

Scenario 1	pemigatinib vs. mFOLFOX						pemigatinib vs. 5-FU					
	Base-case analysis		Probabilistic sensitivity analysis				Base-case analysis		Probabilistic sensitivity analysis			
	ICER (USD/QALY)	INMB (USD)	INMB (USD)		Probability of being cost-effective	EVPI/person (USD)	ICER(USD/QALY)	INMB (USD)	INMB (USD)		Probability of being cost-effective	EVPI/person (USD)
			Mean	95% range					Mean	95% range		
1.1 Base case	80,979	15,736	16,948	(15,798, 18,098)	81.4%	83,475	84,386	16,083	16,878	(15,664, 18,092)	79.9%	2,313
1.2 Life-year as effectiveness	57,454	61,807	64,757	(63,811, 65,702)	100.0%	59,225	57,936	72,360	73,547	(72,593, 74,501)	100.0%	0
1.3 Optimistic: loglogistic distribution	83,581	12,659	13,800	(12,617, 14,983)	75.9%	86,157	86,908	12,911	13,729	(12,480, 14,980)	75.0%	3,201
1.4 Pessimistic: Weibull PH distribution	96,667	-2,289	-2,305	(-3,300, -1,310)	47.1%	99,647	99,589	-2,498	-2,375	(-3,441, -1,310)	48.1%	5,832
1.5 Pessimistic: Weibull PH distribution and 90% price of pemigatinib	87,244	6,270	6,497	(5,563, 7,431)	66.3%	89,932	90,616	6,325	6,427	(5,419, 7,435)	66.5%	4,019
1.6 Time horizon: 2 years	156,004	-27,750	-28,444	(-29,261, -27,627)	0.1%	160,812	152,836	-28,996	-28,851	(-29,742, -27,959)	0.6%	10
1.7 Time horizon: 5 years	103,247	-7,618	-7,720	(-8,700, -6,741)	34.6%	106,428	105,972	-8,125	-7,968	(-9,021, -6,916)	34.4%	3,484
1.8 Time horizon: 10 years	89,830	4,464	4,827	(3,769, 5,886)	61.8%	92,599	93,044	4,440	4,722	(3,593, 5,850)	62.3%	5,374
1.9 Time horizon: 20 years	83,517	12,180	13,025	(11,912, 14,139)	75.4%	86,091	86,879	12,416	12,952	(11,773, 14,133)	74.8%	3,054
1.10 Time horizon: 30 years	81,720	14,669	15,752	(14,614, 16,889)	79.8%	84,238	85,114	14,983	15,681	(14,479, 16,883)	78.5%	2,518
1.11 AE incurred every cycle during first six months	81,489	15,033	16,237	(15,088, 17,388)	80.6%	84,001	86,075	13,665	14,510	(13,295, 15,725)	76.9%	2,833
1.12 AE incurred every cycle	84,102	11,559	12,731	(11,580, 13,882)	74.6%	86,694	89,312	9,285	10,219	(9,002, 11,435)	70.1%	3,986
1.13 AE-specific disutility (event-level incidence)	80,662	16,180	17,404	(16,255, 18,555)	81.8%	83,148	84,063	16,557	17,341	(16,128, 18,556)	80.4%	2,220

Note: Costs are listed in 2023 US dollars. mFOLFOX a combination of oxaliplatin, folinic acid, and fluorouracil; 5-FU/LV fluorouracil and leucovorin; ICER incremental cost-effectiveness ratio; INMB incremental net monetary benefit; EVPI expected value of perfect information; PH proportional hazards; AE adverse event

In Scenario 2, we assessed the marginal effect on CEA results by updating efficacy parameters from the latest FIGHT-202 and NIFTY trial results (Table 4). We first replicated the previous CEA of pemigatinib [9] by adjusting the time horizon, pemigatinib price, and WTP threshold, creating a reference case (Scenario 2.1). The results showed that pemigatinib was not cost-effective, with only a 0.7–1.2% probability of cost-effectiveness using the prior efficacy data. In Scenario 2.2, updating the NIFTY trial for 5-FU/LV yielded ICERs and INMBs similar to Scenario 2.1 with a 0.4% increase in uncertainty. When updating both FIGHT-202 and NIFTY efficacy data (Scenario 2.3), pemigatinib became cost-effective, yielding an expected value of information of US$30,973–US$33,168.

**Table 4:** Scenario and sensitivity analyses: impact of updating efficacy data

Scenario 2	pemigatinib vs. mFOLFOX						pemigatinib vs. 5-FU						
	Base-case analysis		Probabilistic sensitivity analysis				Base-case analysis			Probabilistic sensitivity analysis			
	ICER (USD/QALY)	INMB (USD)	INMB (USD)		Probability of being cost-effective	EVPI/person (USD)	ICER (USD/QALY)		INMB (USD)	INMB (USD)		Probability of being cost-effective	EVPI/person (USD)
			Mean	95% range						Mean	95% range		
2.1 Reference case of scenario 2	134,820	-21,697	-21,071	(-21,582, -20,559)	0.7%	37	127,545		-20,189	-19,505	(-20,092, -18,919)	1.2%	61
2.2 Updated efficacy of 5-FU	134,820	-21,697	-21,071	(-21,582, -20,559)	0.7%	37	131,676		-22,385	-21,691	(-22,302, -21,082)	0.8%	35
2.3 Updated efficacy of 5-FU and pemigatinib	88,309	11,471	11,273	(10,515, 12,031)	82.1%	1,403	89,418		10,784	10,653	(9,806, 11,497)	79.2%	1,990

Note: The reference case in Scenario 2 is set at the listing price of pemigatinib per 13.5 mg, the time horizon of 40 years, and willingness-to-pay threshold of US$97,908 per QALY gain. Costs are listed in 2022 US dollars (1 USD = 29.8 TWD). *mFOLFOX* a combination of oxaliplatin, folinic acid, and fluorouracil; *5-FU/LV* fluorouracil and leucovorin; *ICER* incremental cost-effectiveness ratio; *INMB* incremental net monetary benefit; *EVPI* expected value of perfect information; *AE* adverse event

## Discussion

### Main findings

Our updated CEA, using 50-month efficacy data from the 2024 FIGHT-202 trial, demonstrates that NHIA’s reimbursement for the pemigatinib regimen in patients with advanced ICC and *FGFR2* fusions/rearrangements is cost-effective over a lifetime compared to mFOLFOX or 5-FU/LV, with the INMB reaching the breakeven point in the 8th year. This finding overturns the negative 5-year CEA results and reduces uncertainty of the previous CEA study which was based on the early 2020 trial results at 17.8 months. This shift in CEA results highlights that additional efficacy data and an extended time horizon can significantly affect CEA results and reduce uncertainty in reimbursement decisions, demonstrating a substantial expected value of information.

### Influential CEA parameters

The final results of the 2024 FIGHT-202 trial showed improved outcomes for pemigatinib compared to the 2020 data, highlighting its significant benefit in extending life years and QALYs. In scenario 2 simulations, we applied the 2020 CEA results over a lifetime and updated efficacy with the 2024 trial data. These simulations reveal a substantial difference between using final trial results and earlier midterm data. This underscores the policy implication that withholding drug coverage based on preliminary trial data may deprive patients of innovative treatments and incur opportunity costs.

Our study indicates that pemigatinib would not be cost-effective over short time horizons, such as 2 and 5 years (Scenario 1). Lifetime break-even analysis shows that pemigatinib reaches the breakeven point in the 8th year, a benefit not evident within shorter time horizons. Over 80% of incremental costs of pemigatinib incurred during the PF state. This high initial investment extends PFS by nearly one year and OS by 1.5 years for patients with advanced ICC, compared to 0.25 and 0.39 years with mFOLFOX and 5-FU/LV, respectively. Although pemigatinib’s early PF costs are substantial, the extended survival benefits contribute to a delayed but eventual breakeven. This supports NICE’s recommendation of a lifetime horizon for evaluating treatments with long-term survival benefits [36].

### Implications

In the real world, innovative cancer treatments often receive accelerated approval based on encouraging preliminary results, especially in severe health states or when effective alternatives are lacking. This issue of immature data is particularly critical in advanced therapy medicinal products, such as chimeric antigen receptor T-cell therapy [37]. Balancing the need for speedy accessibility with avoiding the opportunity cost of erroneous decisions due to immature evidence is a significant global challenge.

Our study results carry significant policy implications for provisional payment and reimbursement strategies for innovative medicines with limited evidence. These insights are crucial not only for Taiwan NHIA but also offer valuable perspectives for global health technology assessment bodies. First, Taiwan NHIA’s provisional payment policy is an effective strategy to expedite access to innovative medicines that demonstrate early clinical benefits. This approach meets immediate patient needs even in the absence of comprehensive and robust cost-effectiveness evidence, while ongoing data collection helps HTR to inform future reimbursement decisions. Second, CEA based on early results from pivotal clinical trials should not be solely relied upon for provisional payment decisions. The decision to provide regular coverage requires sufficient and accurate information. CEAs should also align with NICE’s lifetime evaluation criteria [36], as this perspective can overturn short-term CEA findings. This is particularly relevant in cases like ours, where high initial costs for innovative treatments yield substantial long-term survival benefits not captured by short-term analysis. Third, extended data collection after provisional payment should focus on adjusting local parameters and reducing CEA uncertainty. Because advanced ICC is a rare cancer, recruiting sufficient participants within the 5-year provisional payment period is challenging. Thus, instead of aiming for obtaining robust local parameters, using updated pivotal trial data adjusted with local RWD in HTR can be an efficient strategy to update CEA results.

### Limitations

This study has limitations similar to those in our previous work [10, 24, 27, 38, 39], including assumptions about the analytical model approach, study population, and parameter estimations. Without IPD from pivotal trials, we could not estimate transition probability parameters for Markov models, thus unable to assess the model uncertainty of the PSM.

Second, because FIGHT-202 was a single-arm phase 2 trial [8, 16], identifying a common comparator for the ABC-06 [17] and NIFTY [18, 19] trials was not feasible. Consequently, we were unable to estimate indirect hazard ratios to compare the relative efficacy of pemigatinib versus mFOLFOX and 5-FU/LV. Furthermore, lacking IPD prevented us from re-weighting baseline characteristics to adjust for imbalances. As a result, we reconstructed survival curves from published KM curves and conducted a naïve indirect comparison, assuming comparability across the three trial populations. We encourage future research to reevaluate these findings using matching or re-weighting methods once IPD becomes available.

Third, regarding real-world external validation, although pemigatinib has been reimbursed in Taiwan since May 1, 2023, currently available public-use claims data do not yet cover the post-reimbursement period, precluding external validation with local RWD. Regarding AEs, we assumed they occurred in the first cycle, a common approach in oncology CEAs when data on AE timing and frequency are lacking. Notably, the CEA conclusions remained consistent in a scenario analysis testing alternative AE assumptions.

Finally, costs were estimated for patients with advanced ICC using the NHIRD; however, this cohort may not fully align with the trial population due to the lack of clinical examination data in the database. Furthermore, owing to the rarity of cholangiocarcinoma, we identified only 93 patients who met the trial eligibility criteria. Given this limited sample size, we avoided complex statistical adjustments to minimize the risk of overfitting or bias; consequently, differences in baseline characteristics between the cost-estimation sample and the trial population may persist. Nevertheless, although improvements in efficacy observed within the trial period rendered pemigatinib cost-effective, substantial uncertainty remains when extrapolating limited trial-period data to lifetime outcomes. This uncertainty is partly driven by long-tail survival extrapolation, highlighting the need for future health technology assessments to incorporate RWD. We also assumed that non-medication costs for pemigatinib users were identical to those of chemotherapy users, as RWD specifically for pemigatinib are not yet available. Given pemigatinib’s oral administration route compared to the intravenous infusions required for chemotherapy, this assumption likely overestimates costs, resulting in a conservatively higher ICER. Additionally, the NHIRD does not capture out-of-pocket (self-paid) expenses, which may affect cost estimates across different health states. In the absence of specific utility data for advanced ICC, proxy values from other cancer indications were utilized.

## Conclusions

Pemigatinib is cost-effective with the updated efficacy in lifetime simulations for advanced ICC with *FGFR2* fusions/rearrangement with the break-even point at the 8th year, underscoring the value of additional pivotal data and lifetime horizon in HTR. In addition, reassessing cost-effectiveness with the latest pivotal trial data with local RWD can be an efficient method in HTR, particularly for rare cancers, such as advanced ICC. Our findings support Taiwan NHIA’s provisional payment policy as an effective strategy and also offer valuable insights for future reimbursement policy.

## Supplementary Information

Below is the link to the electronic supplementary material.

Supplementary Material 1

## Data Availability

No datasets were generated or analysed during the current study.

## Code availability

All data and software used to construct this model are identified either in the manuscript or in cited references. The R code that supports the findings of this study is available upon request from the corresponding authors.

## Abbreviations

5-FU: Fluorouracil
AE: Treatment-related adverse event
AIC: Akaike information criterion
BIC: Bayesian information criterion
CEA: Cost-effectiveness analysis
CEAC: Cost-effectiveness acceptability curve
DSA: Deterministic sensitivity analysis
EVPI: Expected value of perfect information
FLOFOX: a combination of oxaliplatin, folinic acid, and fluorouracil
GDP: Gross domestic product
HTR: Health technology reassessment
ICC: Intrahepatic cholangiocarcinoma
ICER: Incremental cost-effectiveness ratio
INMB: Incremental net monetary benefit
IPD: Individual patient data
KM: Kaplan–Meier
NHI: National Health Insurance
NHIA: National Health Insurance Administration
NICE: National Institute for Health and Care Excellence
OS: Overall survival
PD: Progressed disease
PF: Progression-free
PFS: Progression-free survival
PSA: Probabilistic sensitivity analysis
QALY: Quality-adjusted life year
RWD: Real-world data
WTP: Willingness-to-pay
