# Supplementary material for: Battle against time for innovative cancer treatment: an updated cost-effectiveness analysis of pemigatinib in intrahepatic cholangiocarcinoma
Source: Cost Eff Resour Alloc. 2026 Feb 10;24:44. doi: 10.1186/s12962-025-00713-w (PMC12990505; doi:10.1186/s12962-025-00713-w)
Supplement: Supplementary file 1 — Supplementary Material 1 [file 12962_2025_713_MOESM1_ESM.docx]

**Supplementary Information**

**Additional File 1**

| Supplementary Table 1: AIC and BIC values in each survival model | | | | | |
| --- | --- | --- | --- | --- | --- |
| Regimen | Distribution | Progression-free survival | | Overall survival | |
|  |  | AIC | BIC | AIC | BIC |
| Pemigatinib | Exponential | 595.4096 | 598.0918 | 658.9657 | 661.6479 |
|  | Weibull | 593.6345 | 598.9987 | 657.7489 | 663.1131 |
|  | Log-normal | 588.8472 | 594.2114 | 654.7308 | 660.0951 |
|  | Log-logistic | 591.552 | 596.9163 | 652.8143 | 658.1786 |
|  | Gompertz | 597.1329 | 602.4971 | 660.6811 | 666.0454 |
|  | Generalized gamma | 590.1901 | 598.2365 | 655.8285 | 663.8749 |
|  | Gamma | 591.7893 | 597.1536 | 656.4546 | 661.8189 |
| 5-FU/LV | Exponential | 386.2919 | 388.7463 | 485.1333 | 487.5876 |
|  | Weibull | 388.2902 | 393.1989 | 480.5723 | 485.481 |
|  | Log-normal | 360.862 | 365.7707 | 463.1411 | 468.0498 |
|  | Log-logistic | 361.5261 | 366.434 | 463.2779 | 468.1866 |
|  | Gompertz | 383.4033 | 388.312 | 486.9945 | 491.9032 |
|  | Generalized gamma | 366.1183 | 373.4813 | 467.0713 | 474.4343 |
|  | Gamma | 386.8752 | 391.7839 | 475.5877 | 480.4964 |

5-FU/LV, fluorouracil/leucovorin; AIC, Akaike information criterion; BIC, Bayesian information criterion
